# Effects of selenium and iodine on Kashin-Beck disease: an updated review

**DOI:** 10.3389/fnut.2024.1402559

**Published:** 2024-05-02

**Authors:** Lin Liu, Pan Luo, Pengfei Wen, Peng Xu

**Affiliations:** Department of Joint Surgery, HongHui Hospital, Xi’an Jiaotong University, Xi’an, China

**Keywords:** Kashin-Beck disease, selenium, iodine, nutrition, thyroid hormone

## Abstract

Kashin-Beck disease (KBD) is an endochondral osteogenesis disorder characterised by epiphysis damage and secondary deformable arthropathy induced by multiple external factors, among which selenium (Se) and iodine deficiency are important influencing factors. Iodine deficiency is usually accompanied by a low Se content in the soil in the KBD areas of China. Se can reverse oxidative damage to chondrocytes. In addition, Se is related to the bone conversion rate and bone mineral density. Low Se will hinder growth and change bone metabolism, resulting in a decrease in the bone conversion rate and bone mineral density. Thyroid hormone imbalance caused by thyroid dysfunction caused by iodine deficiency can damage bone homeostasis. Compared with Se deficiency alone, Se combined with iodine deficiency can reduce the activity of glutathione peroxidase more effectively, which increases the vulnerability of chondrocytes and other target cells to oxidative stress, resulting in chondrocyte death. Clinical studies have shown that supplementation with Se and iodine is helpful for the prevention and treatment of KBD.

## 1 Introduction

Kashin-Beck disease (KBD) is a chronic, endemic, degenerative osteoarthropathy that primarily affects 15 provinces from northeast to southwest China (1). KBD is observed in children and adolescents and is characterised by the necrosis of chondrocytes on the growth plate and articular surface. This pathological manifestation leads to alterations in both the epiphyseal plate and metaphysis, which can lead to growth retardation, bone deformities, joint enlargement, and multiple joint dysfunction (2, 3). The advanced stage of joint deformity, short stature, and even permanent disability in some patients, significantly impair their quality of life (4). KBD is not contagious, and it predominantly occurs in economically disadvantaged and sparsely populated rural areas, typically affecting minority communities (5, 6).

The KBD epidemic in China encompasses the widest geographical area, with the greatest number of patients and highest prevalence rate globally. The statistical data revealed that until the end of 2021, China had 379 endemic counties, affecting approximately 172,000 individuals (7). The incidence of KBD in children has been effectively controlled, and KBD has been nearly eradicated in China; however, in the present study, KBD in adults has remained a significant concern because of its high incidence during the previous century (6). The current clinical measures for repairing cartilage injury or defects in KBD patients are ineffective (6). For example, surgical intervention for abnormal articular cartilage is an efficacious therapeutic approach for patients with KBD exhibiting severe pathological characteristics (8, 9). Considering the current clinical measures, for instance total knee arthroplasty can significantly improve the functionality of KBD patients. The pathological investigation of KBD and the ongoing exploration of effective preventive and therapeutic approaches are still underway.

The regulation and homeostasis of chemical elements are essential for a person’s life, and any disruption to this equilibrium may result in disease onset. Insufficient Se is thought to be closely related to the occurrence of KBD (10). For example, KBD is prevalent in areas with nutritional Se deficiency (11). However, some other evidence was not supportive of this viewpoint. KBD does not manifest in certain areas worldwide despite Se deficiency (12). Moreover, certain regions in China impacted by KBD exhibit average Se levels within their surroundings (13), indicating Se deficiency alone is insufficient to cause KBD. The findings of studies have indicated that children in KBD endemic areas also have insufficient intake of vitamin B2/-C/-E, calcium, iron, zinc, iodine and other nutrients (14). As is widely recognised, iodine is a crucial element essential for the human body (15). Insufficient iodine intake can impair the synthesis, secretion and utilisation of thyroid hormones, affecting bone development and growth plate formation. The occurrence of KBD is attributed to lesions in the articular cartilage and epiphyseal plate cartilage (16). Therefore, iodine deficiency is also considered an important influencing factor of KBD. Moreover, Se deficiency and iodine deficiency mutually influence each other. This review aims to elucidate the roles of Se and iodine in KBD and ascertain the interrelationship among Se, iodine, and KBD.

## 2 Pathogenesis and pathological findings of KBD

### 2.1 Pathogenesis of KBD

**Figure 1:**
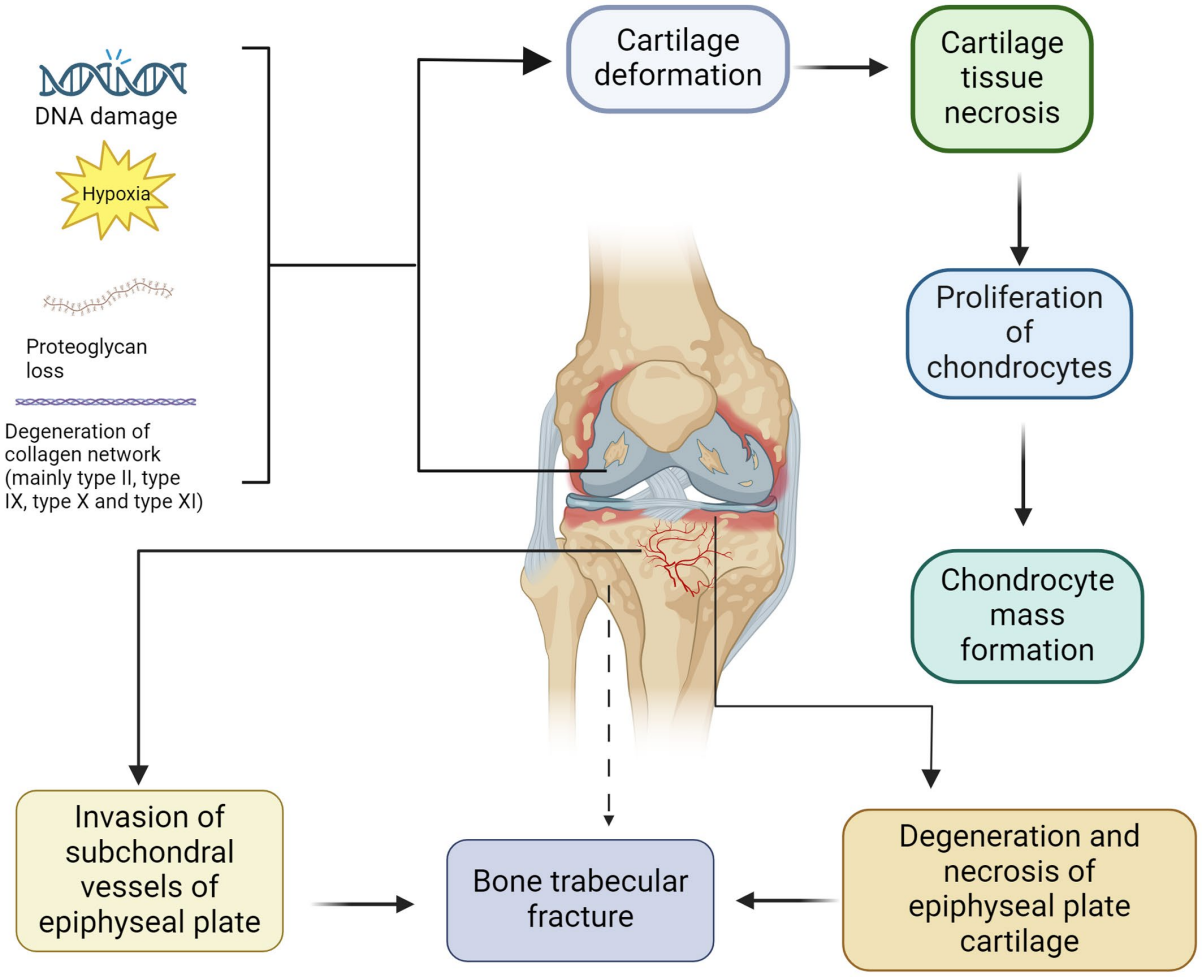
Pathogenesis and pathological manifestations of KBD. The deformation of cartilage and subsequent development of cartilage necrosis can be attributed to DNA damage, hypoxia, loss of proteoglycans and degeneration of collagen network (mainly type II, type IX, type X and type XI). After chondrocyte apoptosis, compensatory hyperplasia of chondrocytes is often present, resulting in the formation of aggregates of proliferating chondrocytes. In contrast, the degeneration and necrosis of epiphyseal plate cartilage can be identical to those of articular cartilage. The small subchondral vessels infiltrate the degenerated and necrotic epiphyseal plate, disrupting the process of ossification and resulting in a reduction in new bone trabeculae density, which increases the risk of fractures.

KBD may arise from the intricate interplay between genetic and environmental factors. Over the years, numerous potential pathogenic factors, exceeding 50 in number, have been proposed by researchers both domestically and internationally. The main hypotheses include the potential involvement of the trace element Se, iodine deficiency, mycotoxin, like T-2 toxin, contamination in grain, and fulvic acid water contamination (17). Various conditions and factors, such as DNA damage, hypoxia, loss of integrin signalling, and activation of death receptors can induce apoptosis activation (18). The analysis of articular cartilage from adult KBD patients revealed the presence of apoptotic chondrocytes in regions exhibiting proteoglycan depletion (19). This finding implies a possible correlation among the depletion of proteoglycans, apoptosis, and disease progression. The extracellular matrix of articular cartilage primarily comprises proteoglycans, which are composed of one or more polysaccharide glycosaminoglycan side chains and a distinctive core protein (20) (Figure 1). The depletion of proteoglycans, particularly aggrecan, in articular cartilage is considered a pivotal initial event in the onset and progression of KBD and represents a target for therapeutic intervention aimed at decelerating disease progression. In addition, collagen also plays an important role in maintaining the function of normal cartilage (21). For example, it can protect the structure of cartilage, maintain the moisture content of cartilage and promote the growth and repair of chondrocytes (22).

Chondrocyte death, including necrosis and apoptosis, can be observed within the articular cartilage of patients diagnosed with KBD (23). Necrosis is a type of traumatic cell death induced by exogenous factors, such as infection, toxins, or physical injury (24). The phenomenon of apoptosis results from a highly regulated process leading to programmed cell death (25). This process is tightly regulated through cellular signalling events, leading to the organised disintegration of individual cells, contraction of the cytoplasm, late loss of cell membrane integrity, and the absence of degradation enzyme release (26).

Bone homeostasis plays a pivotal role in the development, maintenance, and repair of bone tissue. During bone growth and development, bone homeostasis contributes to normal bone development and prevents bone deformation or deformity (27). Osteoblasts have the ability of self-proliferation and self-renewal, which is essential for osteogenic differentiation and bone remodelling (28). The natural expression and apoptosis of osteoblasts are critical for physiological bone homeostasis during bone remodelling. Osteoclasts are bone-resorbing cells derived from haematopoietic stem cells (HSCs) (29). It degrades bone via the secretion of acid and proteolytic enzymes, such as cathepsin K, which dissolve collagen and other matrix proteins during bone resorption (30). Studies have shown that osteoblasts and osteoclasts can communicate with each other through direct cell-to-cell contact and cytokine and extracellular matrix interactions (31, 32). The bone remodelling process involves replacing old or damaged bone with new bone through a series of cellular events occurring on the same surface while maintaining the original shape of the bone (33). In addition, this process is also elementary way to reshape the bone. This inference suggests a potential association between KBD and impaired bone homeostasis.

### 2.2 Pathological findings of KBD

The primary pathomorphological changes in KBD primarily manifest in the endochondral bone, particularly affecting the joints of the extremities. Except for the sternocleidomastoid and mandibular joints, all other joints may exhibit varying degrees of involvement (34, 35). KBD is characterised by dystrophic and degenerative changes in cartilage, constituting its pathological essence. The primary manifestations include degeneration, necrosis, and atrophy of the hyaline cartilage components within the endochondral osteoid framework, including epiphyseal cartilage, epiphyseal plate cartilage, and articular cartilage (6). Moreover, repairing and adaptive changes lead to epiphyseal endochondral osteogenesis disorders and secondary degenerative arthropathy (36). When the self-repairing function of bone tissue is impaired, fractures or trabecular fractures may occur, leading to the formation of small cystic spaces (37).

#### 2.2.1 Basic cartilage lesions

The characteristic pathological changes of KBD include the demise of articular chondrocytes within the cartilage matrix. Additional pathological alterations include fibrofibrillation on the surface of the cartilage and the formation of aggregates comprising chondrocytes (38, 39). The most significant pathological alterations in KBD involve the degeneration and necrosis of cartilage. The manifestations of chondrosclerosis include the presence of a lightly red-stained cartilage matrix, mucinous degeneration, a fibrillar appearance, asbestoid degeneration, fissure formation, and necrosis of chondrocytes and cartilage tissue (40). The secondary changes following chondronecrosis are characterised by local tissue disintegration and liquefaction, hyperplasia of chondrocytes surrounding the necrotic lesion, and the formation of masses of chondrocytes (38).

The processes of absorption, removal, calcification, organisation, and ossification after cartilage necrosis are all integral components of the reparative mechanism (41, 42). The bone tissue formed due to cartilage necrosis does not meet the physiological and mechanical requirements of a normal skeleton in terms of arrangement and quantity; thus, it is referred to as “scar bone tissue” (42).

#### 2.2.2 Lesions of epiphyseal plate cartilage

Unlike osteoarthritis, in juvenile patients, the epiphyseal plate cartilage is more prominent and susceptible to degeneration and necrosis, similar to that of articular cartilage. The invasion of small subchondral blood vessels into the degenerated and necrotic epiphyseal plate leads to osteoclast dissolution, resorption, and irregular new bone tissue formation, thereby inducing early ossification (43). Due to the disorder of the ossification process, newly formed bone trabeculae are not only sparse and short but also exhibit a change in direction from a normal longitudinal arrangement to a transverse orientation, thereby obstructing longitudinal bone growth (44).

## 3 Selenium and Kashin-Beck disease

### 3.1 Selenium

The trace element Se is crucial and widely distributed across the Earth’s atmosphere, lithosphere, and hydrosphere. The predominant forms of Se in the natural environment are inorganic compounds, including selenite, selenide, and selenate (45). Moreover, Se is an indispensable trace element for the human body, exhibiting diverse biological functionalities. It actively participates in numerous physiological processes, including immune modulation and antioxidant defence mechanisms (46). Se activates antitumour factors, mitigates cardiovascular diseases, exhibits antiproliferative and anti-inflammatory properties, and enhances immune system stimulation (47). Se deficiency can contribute to the development of endocrine and immune disorders, infections, chronic inflammation, neurodegeneration and cardiovascular disease, impacting life expectancy (48).

The antioxidant properties of Se make it a valuable dietary supplement that is widely recognised for its potential to promote health, prevent disease, and slow the ageing process (49). Se supplementation has been shown to protect against various detrimental factors, including chemical agents such as severe drug side effects, heavy metals, carcinogens, mycotoxins or pesticides, and physical stressors such as heat or magnetic fields (50). The inclusion of organic Se in food is widely acknowledged as a safe and efficacious means of supplementing human, animal, plant, and mushroom diets with Se. The primary dietary sources of Se include red meat, poultry, organ meats, seafood, eggs, and dairy products (51, 52). Despite being recognised as a dietary supplement for enhancing health, Se exhibits a narrow safety margin between deficiency and overdose, with the potential for toxic effects upon excessive intake. Se toxicity occurs when the intake surpasses the human tolerance threshold of 400 μg/day (53).

### 3.2 Impact of Se on the pathogenesis of KBD

**Figure 2:**
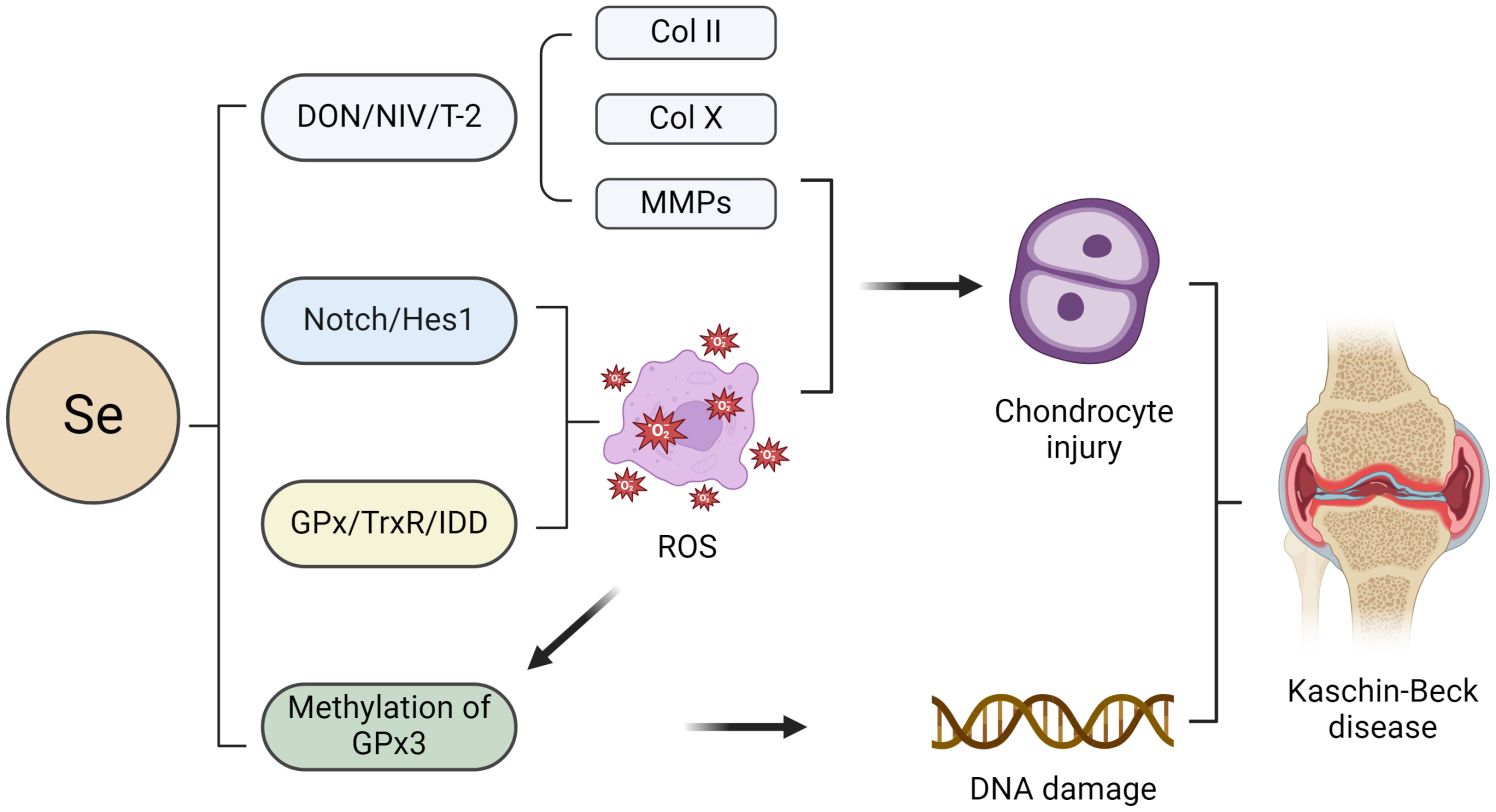
Impact of selenium on chondrocytes in the pathogenesis of KBD. The combination of selenium deficiency with deoxynivalenol (DON), nivalenol (NIV), and T-2 leads to chondrocyte injury characterised by the loss of aggrecan and type II collagen (COLII) as well as an increase in the expression of COLX and matrix metalloproteinases (MMPs). Activation of the Notch/Hes1 signalling pathway may lead to excessive apoptosis of chondrocytes, potentially contributing to selenium deficiency-induced effects. The primary role of several selenium proteins, including glutathione peroxidase (GPx), thioredoxin reductase (TrxR), and iodothyronine deiodinase (DIO), is to mitigate oxidative damage in chondrocytes as crucial intracellular antioxidants. Oxidative stress-induced GPx3 methylation may contribute to the pathogenesis of KBD by inhibiting GPx3 expression.

Se exhibits the second highest concentration in bone, followed by skeletal muscle (54). The results of a meta-analysis indicated that Se levels in water, soil, grain, and corn were significantly lower in KBD-endemic areas than in nonendemic areas. These findings imply that tissue Se levels are predominantly influenced by dietary consumption (55). Se, an indispensable trace element, exerts its biological functions by forming Se proteins intricately involved in diverse physiological processes, including redox homeostasis, anti-inflammatory responses, and thyroid hormone metabolism (56). Se is an essential component of various enzymes and proteins, of which at least nine Se proteins are involved in the natural expression of osteoblasts (57). Several Se-containing proteins, including glutathione peroxidase (GPx), thioredoxin reductase (TrxR), and iodothyronine deiodinase (DIO), primarily function as crucial intracellular antioxidants to mitigate oxidative damage (58). Under conditions of Se deficiency, target cells such as chondrocytes experience oxidative stress, leading to necrosis of the growth plate and indirectly impacting cartilage development (59) (Figure 2). Numerous studies have demonstrated that the pathophysiology of KBD involves excessive chondrocyte apoptosis and oxidative stress. However, the precise underlying mechanisms remain elusive (60, 61).

Previous studies have demonstrated an association between Se protein-encoding gene polymorphisms and heightened susceptibility to KBD. For instance, the mRNA expression level of GPX1 and enzymatic activity of total GPX in the blood of KBD patients were lower than those of controls (62). In addition, Yang et al. found that the mRNA level and GPX activity of GPX3 were downregulated in patients with Se deficiency, indicating that the selenoprotein transcriptional spectrum was out of order in patients with KBD (63). The TCC, TTC, and TTT haplotypes of rs1050450, rs3811699, and rs1800668 in the GPX1 gene are also significantly linked to KBD (64). In addition, Se deficiency impaired bone and cartilage growth, resulting in premature chondrocyte hypertrophy, as evidenced by increased COLX expression, which is compatible with the phenotypes of KBD cartilage (65). Epigenetics offers the most comprehensive framework for investigating the underlying mechanisms by which environmental factors contribute to the development of KBD. The regulation of gene expression in various diseases is governed by epigenetic modifications, particularly DNA methylation, in response to environmental exposures (66, 67). DNA methylation hinders the recruitment of transcription factors, impeding the transcription of diverse genes and rendering them dysfunctional. Se has been implicated in epigenetic modifications in numerous studies (68, 69). GPx3, a pivotal Se-containing protein within the glutathione peroxidase family, is the principal antioxidant enzyme in the human body. In a study investigating GPx3 in KBD patients, the methylation level of GPx3 in whole blood samples from KBD patients was significantly greater than that in whole blood samples from healthy controls (70). Oxidative stress-induced epigenetic modification of GPx3 may exert regulatory control over the transcription of antioxidant genes and mRNA stability. It can be inferred that GPx3 methylation may lead to the inhibition of GPx3 expression, potentially contributing to the pathogenesis of KBD. Nevertheless, DNA methylation is reversible, and the restoration of GPx3 function can be achieved through methylation removal (70). Se supplementation enhanced GPx3 mRNA expression and attenuated GPx3 methylation.

Low-Se conditions in combination with three mycotoxins, deoxynivalenol (DON), nivalenol (NIV), and T-2, induced pro-catabolic changes and a hypertrophic phenotype in chondrocytes, as evidenced by the loss of aggrecan and type II collagen (COLII) and the increase in COLX and matrix metalloproteinase (MMP) expression, respectively (71). In contrast, Se supplementation partially alleviated this mycotoxin-induced damage in chondrocytes (71). In this case, epiphyseal plate lesions in KBD patients can be observed, and the expression of COLII and GPX1 in chondrocytes is reduced, indicating a potential association between the observed epiphyseal plate lesions in KBD patients and decreased chondrocyte anabolism, metabolism, and antioxidant capacity (72). Additionally, Zhang et al. reported a potential correlation between the reduced plasma Se levels observed in KBD patients and inadequate dietary Se intake (73). Se deficiency may be involved in the pathological process of KBD by activating the Notch/Hes1 signalling pathway, leading to excessive chondrocyte apoptosis. Activation of Notch/Hes1 promotes oxidative damage, while supplementation with Se can reverse this oxidative damage (73). These findings suggest that targeting the Notch/Hes1 signalling pathway may be a novel therapeutic approach for the management of KBD.

The pathogenesis of bone remodelling disorders in KBD patients is attributed to an imbalance between osteoblast-mediated bone formation and osteoclast-mediated bone resorption (74). The relationships between Se and the bone turnover rate and bone mineral density have been established by various studies (75–77). Inadequate Se levels can impede growth and alter bone metabolism, decreasing the bone turnover rate and bone mineral density (78). The expression of Se proteins in human foetal osteoblasts protects against oxidative stress-induced bone damage. The impairment of osteoblast differentiation from bone marrow stromal cells may contribute to the pathogenesis of low bone density (79). The expression of GPx and TrxR in bone marrow stromal cells cultured with a low Se concentration was suboptimal and accompanied by chromosomal damage (80). These phenomena could be ameliorated through Se supplementation. The activity of the Se protein was also restored upon supplementation with Se (80). The protective effect of Se against bone loss has also been demonstrated in a murine model of cadmium-induced bone loss. The administration of sodium selenite, an inorganic Se compound, effectively restores the activity of antioxidant enzymes within the skeletal system and facilitates the restoration of bone mass (81).

## 4 Iodine and Kashin-Beck disease

### 4.1 Iodine

Iodine, a trace element predominantly concentrated in or near coastal regions, is abundant in the Earth’s crust. The essentiality of iodine lies in its regulation of various pivotal physiological functions, including metabolism and brain development. Iodine plays a crucial role in various biological functions. Recent research has revealed the antioxidant, antibacterial, and antitumour properties of iodine; however, its most prominent and well-established function within the human metabolic pathway lies in the physiology of the thyroid gland (82). Iodine serves as the fundamental constituent of thyroid hormones (83). A healthy adult typically possesses 20–50 mg of iodine within their body, with approximately 70–80% predominantly localised in the thyroid gland, where it serves as an essential constituent for the synthesis of thyroid hormones (84). Iodine is rapidly absorbed from the gastrointestinal tract and subsequently primarily eliminated via renal excretion, with only a minor fraction excreted through perspiration, saliva, lacrimation, and biliary secretion. The urinary iodine concentration is a highly reliable parameter for assessing iodine intake (85, 86).

Due to insufficient iodine content in food, various dietary habits may increase the risk of inadequate iodine intake, particularly among vegetarians. The global prevalence of iodine deficiency disorders constitutes a significant public health challenge, affecting approximately two billion individuals worldwide. Hypothyroidism resulting from iodine deficiency can have detrimental effects on the skeletal, muscular, and reproductive systems (87, 88). The effects of iodine deficiency can impact individuals of all age groups, with pregnant women especially susceptible to its health implications (89). In numerous nations where iodine prevention initiatives are being implemented, using iodised salt is a pivotal strategy for augmenting iodine levels in households and the food industry, as is incorporating dietary supplements. The Global Salt Iodisation Program was implemented in the 20th century to enhance worldwide iodine consumption, marking the initiation of efforts against iodine deficiency (90).

### 4.2 Impact of iodine on the pathogenesis of KBD

The occurrence of KBD has been frequently observed in regions with a high prevalence of iodine deficiency disorders, as indicated by epidemiological investigations (15, 91). The influence of the endocrine system on KBD has long been acknowledged by researchers in the field. The impact of thyroid function on KBD has been extensively investigated both domestically and internationally (92). However, the findings have exhibited incongruity. The data indicate that both hypothyroidism and hyperthyroidism affect KBD. In a 1998 study conducted in Tibet, Moreno-Reyes et al. discovered that individuals with lower urinary iodine concentrations and higher serum thyrotropin concentrations were at an elevated risk of developing KBD (15). It can be inferred that hypothyroidism caused by iodine deficiency plays a substantial role in influencing KBD within rural regions of Southwest China.

The thyroid gland, one of the most crucial endocrine organs, regulates physiological processes through the synthesis of thyroid hormones and secretion of calcitonin. The thyroid gland accumulates the essential trace element iodine as an inorganic anion known as iodide. Iodine deficiency is a prevalent cause of hypothyroidism in humans and subsequently impacts bone growth and development (93). Thyroid hormones are crucial in facilitating the normal growth, differentiation, and physiological functioning of diverse tissues (94).

**Figure 3:**
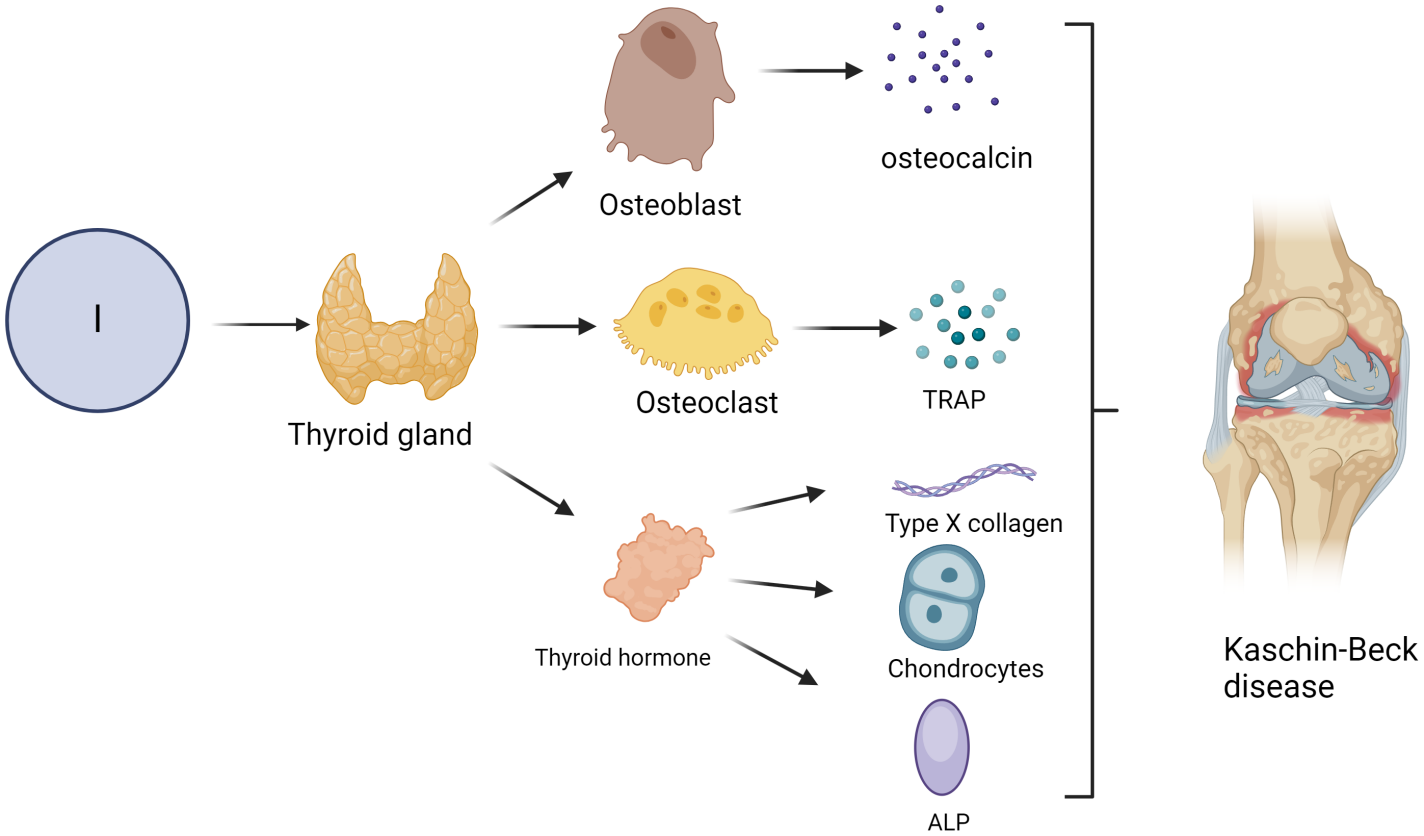
Impact of iodine deficiency on the incidence of KBD. The occurrence of KBD may be attributed to hypothyroidism resulting from iodine deficiency. Thyroid hormones, for instance, can induce the expression of type X collagen and alkaline phosphatase (ALP), facilitate the proliferation and terminal differentiation of growth plate chondrocytes, and promote endochondral ossification. Furthermore, thyroid hormone promotes the expression of osteocalcin and facilitates bone formation while also promoting the differentiation of osteoblasts and inhibiting their proliferation. Thyroid hormones can stimulate the expression of tartrate-resistant acid phosphatase (TRAP) and osteoclast formation, stimulate osteoclast differentiation and maintain bone mass.

Disruption of the thyroid hormone balance caused by a dysfunctional thyroid can compromise bone homeostasis. Hypothyroidism impedes the process of bone remodelling (95), resulting in diminished stature, delayed skeletal maturation, abnormalities in epiphyseal development, and postponed dental growth. The pathogenesis of hypothyroidism involves impaired osteoblast-mediated bone formation and enhanced osteoclast-driven bone resorption, leading to a prolonged duration of the bone remodelling cycle. Extended cycles of bone remodelling involve extended periods of secondary mineralisation, resulting in increased bone mineralisation and mass without any changes in bone volume (96, 97). In contrast to hypothyroidism, hyperthyroidism is associated with an increased bone turnover rate, an increased frequency of bone remodelling sites, and an accelerated rate of both bone resorption and formation, leading to a decline in bone mass and reduced bone mineral density (98, 99). An imbalance between bone resorption and formation, coupled with a shortened remodelling time, results in an approximate 10% loss of bone mass during each cycle of bone remodelling. The presence of hyperthyroidism can result in the development of osteoporosis and increased susceptibility to fractures. Furthermore, the literature has indicated that iodine deficiency can diminish antioxidant capacity (100). The antioxidant properties of iodine may significantly mitigate the damage caused by KBD, as per the free radical damage mechanism associated with this condition. The T4 level in rats with iodine deficiency was significantly lower than that in the normal control group. Additionally, there is a decrease in bone length and thickness, smaller secondary ossification centre, disordered growth plate chondrocytes, a reduced number of reserve chondrocytes and width of the proliferation zone, a significant reduction in trabecular bone volume and thickness, decreased osteoblast activity, and an impaired bone mineralisation process (101). Iodine deficiency can also impair bone growth. Thyroid hormones stimulate the expression of type X collagen and alkaline phosphatase (ALP), promote the proliferation and terminal differentiation of growth plate chondrocytes, and facilitate the formation of the cartilage endoskeleton (102) (Figure 3). The impact on osteoblasts is demonstrated by stimulating the expression of osteocalcin and promoting bone formation, facilitating osteoblast differentiation, suppressing osteoblast proliferation, and contributing to the maintenance of bone mass. The effect on osteoclasts is evidenced by an increase in tartrate-resistant acid phosphatase (TRAP) expression and the promotion of osteoclastogenesis, facilitating the differentiation of osteoclasts and aiding in the preservation of bone density (103–105).

## 5 Mechanism underlying the interaction between Se and iodine in relation to KBD

The interaction between Se deficiency and iodine deficiency is mutually influential. Most areas affected by KBD have both Se and iodine deficiencies. Ren et al. reported that insufficient Se and iodine adversely affect bone and cartilage development (65). The alterations in the expression of *ColX* and *PTHrP* resulting from concurrent Se and iodine deficiency were consistent with those observed in KBD (65). The primary mechanism underlying the interaction between Se and iodine lies in the synthesis and subsequent utilisation of thyroid hormones. The presence of Se is closely linked to the function of peroxidases in the synthesis of thyroid hormones. Glutathione peroxidase plays a crucial role in safeguarding the thyroid gland against the detrimental effects of hydrogen peroxide (106). Se is an important component of antioxidant defences, and Se deficiency may exacerbate the damage caused by iodine deficiency to the thyroid (107). Iodothyronine deiodinase can initiate or terminate thyroid hormone activity, dynamically regulating thyroid hormone signal transduction (108). Activation of T4 is catalysed by the types 1 and 2 iodothyronine deiodinases (referred to as D1 and D2) while the inactivation of T4 and T3 is the function of a third deiodinase, the type 3 deiodinase (D3) (109). The presence of deiodinases is indispensable for the physiological effects mediated by thyroid hormones. Se deficiency impacts the activity or expression of deiodinase, influencing thyroid hormone metabolism. Se in selenoproteins is present as Se-cysteine in the active centre, enabling it to perform its biological functions effectively (110). Approximately 30 distinct types of Se-containing proteins have been identified, with 25 such proteins being discovered within the human body (111). The glutathione peroxidase family, the proline deiodinase family, thioredoxin reductase, and Se phosphate synthetase are pivotal in human physiology (58). Se deficiency leads to abnormal thyroid hormone metabolism through the type 1 thyroid hormone proto-deiodinase and reduces glutathione peroxidase activity (112).

Both Se and iodine play crucial roles in maintaining the normal function of thyroid hormones (113). Compared with simple Se deficiency, concurrent Se and iodine deficiency further diminishes the activity of glutathione peroxidase, increasing the vulnerability of target cells, such as chondrocytes, to oxidative stress and leading to chondrocyte apoptosis (114). Moreover, Se supplementation may expedite iodine metabolism in instances of insufficient iodine intake (113). This leads to the deiodination of circulating thyroid hormones, accelerating iodine loss in the kidney and faeces and exacerbating iodine deficiency (113). The administration of Se supplements, whether preventive or interventional, should be preceded by ensuring adequate iodine levels to prevent iodine loss. In conclusion, Se and iodine deficiencies are environmental pathogenic factors in the development of KBD, and their roles in maintaining antioxidant levels and thyroid function may be crucial for KBD.

## 6 Prevention and treatment of KBD

KBD imposes a substantial burden on patients, their families, and society at large. Therefore, it is imperative to implement effective intervention measures to prevent and control KBD. Over the past four decades, Chinese public health organisations have implemented comprehensive prevention and control measures in endemic regions, significantly reducing both new cases and the prevalence of KBD (115). However, the prevalence remains high in western China, particularly in Sichuan, Qinghai and Xizang (7). Given that T-2 toxin pollution is the most extensively studied form of food contamination in KBD regions (116), it is imperative to focus on elucidating the underlying causes of T-2 toxin accumulation in food and developing effective strategies for its control and mitigation as crucial measures to prevent KBD. The KBD region is characterised by low temperatures and high humidity levels (117). Reckless practices such as grain planting, harvesting, and processing increase the probability of T-2 toxin dissemination. Additionally, high humidity and inadequate sanitation during storage may contribute to increased production of T-2 toxin. The storage environment should, therefore, be enhanced, encompassing improvements in sanitary conditions, the reinforcement of ventilation systems, and a reduction in the quantity of stored wheat flour.

Although there are various methods, such as nonsteroidal anti-inflammatory drugs and steroids (118), hyaluronan injections (119), Se supplementation (120), physical therapy (121) and various techniques for joint debridement (122), these methods may provide short-term relief of KBD symptoms, and they cannot repair cartilage damage or halt disease progression. Joint defects have been effectively treated through surgical interventions by orthopaedic surgeons from China (123, 124). The supplementation of Se salts in the diet is considered a potential approach for the prevention and treatment of KBD. Currently, oral sodium selenite is the primary methods used for Se supplementation (125). Chondroitin sulfate nano-selenium has been tested as a supplement of selenium (126). Se supplementation has a significant impact on the treatment of KBD. For instance, it can effectively facilitate the repair process of metaphyseal lesions and delay cartilage degradation (50). In an investigation of the impact of five different Se supplementation approaches on the treatment of KBD in paediatric patients, all the Se supplementation methods exhibited superior efficacy compared with that of a placebo or no treatment in repairing metaphyseal lesions (2). However, evidence from recent systematic reviews and randomised controlled trials suggests that Se supplementation (typically 200 micrograms per day) increases the risk of diabetes (127). Therefore, we should pay attention to not exceeding the recommended daily dose of Se. Some agencies have recently lowered their daily Se intake limits. For example, the European Food Safety Agency says that the maximum tolerable intake for adult men and women, including pregnant and lactating women, is 255 μg per day. Current research is focused on the development of diverse Se-based therapeutic strategies rather than traditional Se intake tests. Researchers have conducted experiments using synthetic green Se nanoparticles, which have been shown to inhibit osteoclastogenesis while promoting osteoblastogenesis (128). Fatima et al. also discovered that moderate concentrations of Se nanoparticles can enhance the survival and osteogenic potential of human mesenchymal stem cells by mitigating oxidative stress (129). Qiao et al. found that chondroitin sulfate nano-selenium supplement increased the number of living chondrocytes, improved the ultrastructure, and altered the expressions of chondroitin sulfate structure-modifying sulfotransferases, Caspase-9, and Cyt-C (126). However, the literature is scarce on the green synthesis of Se nanoparticles, necessitating further investigation into the interplay between Se and Se nanoparticles, as well as elucidation of the underlying biological pathways responsible for their differential therapeutic efficacy.

In addition to Se supplementation, researchers emphasise the significance of considering holistic nutrition rather than solely focusing on Se. For example, Ning et al. reported that the intake of fat, carotene, retinol, multivitamins, dietary fibre and trace elements in children in endemic areas was still significantly lower than that in children in nonendemic areas (130). Furthermore, the dietary composition is characterised by its limited variety, rendering it insufficient to meet the recommended intake of essential nutrients (130). In another cross-sectional study conducted in Shaanxi Province, an endemic area of KBD, it was observed that consuming grains, meat, and milk was crucial in enhancing Se intake among children (131). In summary, the lack of selenium intake in children in endemic areas may be due to the lack of diversified dietary structures, resulting in limited intake of selenium-rich foods such as meat, eggs, and bean products. Therefore, we need to take measures such as nutrition education to optimise the dietary structure of residents in epidemic areas and correct the imbalance of children’s comprehensive nutrient intake.

## 7 Conclusion

Despite significant advancements in research on the aetiology and pathogenesis of KBD, numerous uncertainties regarding its aetiology and pathogenesis necessitate further investigation and discussion. The role of Se in the pathogenesis of KBD remains unclear, and its association with certain aspects rather than direct causation of the disease has been suggested. The impact of Se on KBD and bone health remains a burgeoning area of research, necessitating further investigation and practical application in the future. However, the paucity of research investigating the association between iodine and KBD necessitates further investigation and analysis. The occurrence of KBD is attributed to either a single environmental factor or a combination of multiple environmental factors, and the intricate interplay between primary and secondary issues necessitates extensive investigations for comprehensive analysis.

## Author contributions

LL: Conceptualization, Methodology, Project administration, Resources, Software, Validation, Writing – original draft, Writing – review & editing. PL: Conceptualization, Data curation, Investigation, Methodology, Validation, Writing – original draft, Writing – review & editing. PW: Conceptualization, Data curation, Investigation, Methodology, Project administration, Resources, Software, Supervision, Writing – original draft, Writing – review & editing. PX: Conceptualization, Funding acquisition, Investigation, Methodology, Project administration, Supervision, Validation, Visualization, Writing – original draft, Writing – review & editing.
